# The clinical effect of an electric massage chair on chemotherapy-induced nausea and vomiting in cancer patients: randomized phase II cross-over trial

**DOI:** 10.1186/s12906-024-04464-8

**Published:** 2024-04-19

**Authors:** Ju Won Kim, Ah Reum Lim, Ji Yoon Lee, June Young Lee, Soohyeon Lee, Yoon Ji Choi, Yeol Hong Kim, Kyong Hwa Park

**Affiliations:** 1 Division of Oncology/Hematology, Department of Internal Medicine, Korea University Anam Hospital, 73 Goryeodae-ro Seongbuk-gu, Seoul, 02841 South Korea; 2 Korea University Anam Hospital, 73 Goryeodae-ro Seongbuk-gu, Seoul, 02841 South Korea

**Keywords:** CINV, Massage, Massage chair, Palliative care, Integrative medicine, Complementary medicine

## Abstract

**Purpose:**

Chemotherapy-induced nausea and vomiting (CINV) is a common adverse events in cancer patients and can negatively affect their quality of life (QoL). This study aimed to evaluate the clinical efficacy of an electric massage chair (EMC) for the treatment of CINV.

**Methods:**

A randomized phase II cross-over trial was conducted on solid cancer patients who received moderate (MEC) to high emetogenic chemotherapy (HEC). The participants were randomly assigned to receive their first chemotherapy either on a standard bed (Group A) or in an EMC (Group B) during the infusion. The patients were then crossed over to the next cycle. CINV and QoL questionnaires were collected from the participants.

**Results:**

A total of 59 patients completed the trial protocol and were included in the analysis, with 29 and 30 patients in Groups A and B, respectively. The mean INVR (Index of Nausea, Vomiting, and Retching) score in the 2nd day of the first cycle was higher in Group B (3.63 ± 5.35) than Group A (2.76 ± 4.78), but the difference was not statistically significant (*p* = 0.5367). The complete response rate showed little difference between the groups. Among the high-emetic risk subgroups, patients who received HEC (*p* = 0.04595), younger patients (*p* = 0.0108), and non-colorectal cancer patients (*p* = 0.0495) presented significantly lower CINV scores when EMC was applied.

**Conclusion:**

Overall, there was no significant difference in INVR scores between standard care and EMC. Applying EMC at the first chemotherapy infusion may help preserve QoL and reduce CINV in high-risk patients.

**Trial registration:**

KCT0008200, 17/02/2023, Retrospectively registered.

**Supplementary Information:**

The online version contains supplementary material available at 10.1186/s12906-024-04464-8.

## Introduction

Chemotherapy-induced nausea and vomiting (CINV) significantly impacts the quality of life (QoL) of cancer patients and is a major side effect of chemotherapy. The pathophysiology of CINV involves the peripheral and central nervous system, with acute CINV triggered by serotonin release through chemotherapeutic agents, and delayed CINV involving substance P [1–4]. Chemotherapeutic agents are categorized by emetogenic potential: high (> 90% incidence of CINV), moderate (30–90%), low (10–30%), and minimal (< 10%) emetic risks [5]. Despite advancements in antiemetics, CINV prevention is not fully achievable, and side effects like constipation and dizziness remain issues [6].

Various alternative treatments and rescue medications have been explored to alleviate CINV [7, 8]. Among non-pharmacological therapies, massage therapy has been investigated as a non-invasive option and has shown benefits in reducing depression, stress, anxiety, and nausea in cancer patients [9]. As an alternative to human massage therapists, mechanical devices are becoming increasingly popular [10], driven by the need for healing and comfort in modern society. The surge in caregiving service demand, due to the rise in single-person households and healthcare labor costs, has led to a push for replacing cancer care for palliation with machines. Among these mechanical options, the electric massage chair (EMC) is designed to automatically massage the user’s entire body using digital and mechanical tools. Additionally, modern EMCs offer mental care through natural sounds and relaxing music. A previous study found that combining soothing massage and music therapy effectively reduced physical/mental fatigue and enhanced cognitive function in healthy adults [11–14].

Given this background, administering chemotherapy in an EMC could positively impact distressed cancer patients, particularly those suffering from CINV. However, the clinical efficacy of EMC remains largely unexplored. This study was conducted as an open-label, randomized trial to assess the effectiveness of an EMC in preventing CINV in cancer patients.

## Methods

### Study design

#### Sample size calculation

Under both a significance level of 5% and a power of 80%, the number of patients required to show that the proportion of patients reaching an INVR (Rhodes Index of Nausea, Vomiting, and Retching) score of less than 9 increased by 35% when using an EMC was at least 30 patients per group (60 patients in total). The expected reach rate of 75% for the group using the EMC and 40% for the group not using the EMC was assumed, and this 35% difference was assumed to be the minimal clinically important difference. Assuming a 10% dropout rate, 68 patients (34 patients per group) were planned.

#### Study protocol

This study was designed as an open-label, phase 2 clinical trial conducted at the Korea University Anam Hospital. Between June 2020 and July 2021, solid cancer patients scheduled to receive HEC or MEC for the first time in their life were screened. Those who had disseminated or extensive bone metastasis or were taking anticoagulant agents were excluded. To prevent bias, patients already using EMC at home were also excluded. When the patients met all the inclusion and exclusion criteria, they were enrolled in the study and randomized into two groups: Group A or Group B. The randomization process was conducted independently by the research team using computer software that generated the random sequence. Patients in Group A received chemotherapy on a general ward bed, while those in Group B received chemotherapy in an EMC during the first cycle of treatment. At the 2nd cycle of chemotherapy, participants crossed the other group. We assumed that at least 10 days after receiving an EMC treatment was enough to wash out the clinical effect of massage, and that there might be no carry-over effect on the next cycle of chemotherapy. Standard prophylactic antiemetics were prescribed according to each chemotherapy regimen, and rescue medicines were also added according to patients’ needs and investigators’ decisions. Acute CINV was evaluated on the second day of each chemotherapy cycle according to the CTCAE v5.0 [9]. Information regarding rescue medication for CINV and QoL surveillance was also collected. The study protocol was reviewed and approved by the Institutional Review Board of Korea University Anam Hospital (No. 2020AN0054).

#### Goal of the study

Primary endpoint of the study was an INVR score for two groups in the acute (0–24 h) phase of chemotherapy. Secondary endpoints included complete response (CR: no emesis, no rescue medication) rate during the acute (0–24 h) and delayed (24–120 h) phase defined by previous study [15], and QoL scale assessed by EORTC-QLQ-C30.

### The intervention – EMC

The intervention group underwent mechanical massage using automatic EMCs (Phantom Medical, BFR-M8030, Bodyfriend Inc., Seoul, Korea). The EMC was approved as a medical device certified by the Ministry of Food and Drug Safety and induced muscle relaxation through whole-body massage, including the neck, shoulders, arms, hands, waist, hip, calf, and soles of the feet. Physical force was applied to the soft tissue of the body through massage modules, calf and foot rollers, and airbags. The device was equipped with medical modes with up to 38 kneading and 560 tapping functions per minute of massage.

In the present study, the massage module was set to gently knead the soft tissue of the body around the neck and back during the session. Leg massage was provided through calf and foot rollers and airbags at mild intensity. Each massage session lasted of a total of 20 min. Initially, it offers neck and shoulder kneading, rolling massage, and comforting music for muscle relaxation and stability over the first 5 min. Then, for 10 min, it provides bilateral stimulation and a narrative approach with psychiatric healing messages, aimed at transforming negative emotions into positive ones. The final 5 min focus on soothing the entire body to relax tense muscles.

The intervention group received at least one massage session per day. Unless side effects were reported, the massage session was continued for another 20 min. Reclining and tilting of the massage chair were included to enhance the effect of the mechanical massage. Upper extremity airbags were provided only in the arm where the intravenous line was not administered.

Along with physical massage, the EMC also provided audio to the patients. Comforting New Age-style music without lyrics followed by a narrative message of hope and courage, orchestrated by a psychiatrist, was presented during the intervention.

### Assessment of CINV

The degree of acute CINV was measured on the second day after chemotherapy initiation. To assess the severity of acute CINV, we adopted the Korean version of the INVR, which was translated from the English version and validated previously [10]. The INVR scores range from 0 to 32 and the higher the score, the greater the severity of nausea and vomiting. Along with INVR, we also collected the patients’ subjective CINV rating scales. The CINV score ranged from 0 (no symptoms) to 10 (worst imaginable symptoms). A higher score indicated greater CINV intensity. For the assessment CINV, we measured timepoints based on guidelines established in previous studies [15]: the acute phase (0–24 h) and the delayed phase (24–120 h). Additionally, following the protocol of the two-week interval triplet chemotherapy, we conducted another check on day 10.

After chemotherapy, rescue medication was permitted to treat refractory CINV and the timing of rescue medicine was collected by the study coordinator to calculate the CR. Information on rescue medication in the acute phase (0–24 h) was collected using the electronic prescription system. Information during the delayed phase was self-reported by the patients.

### QoL assessment

Patient-reported outcomes were obtained before starting chemotherapy, the day before the starting 2nd cycle (completing the 1st cycle), and the day before starting the 3rd cycle (completing 2nd cycle). We adopted the Korean version of the EORTC-QLQ-C30 to assess patient QoL. The EORTC-QLQ-C30 is a survey form that incorporates a 30-item cancer-specific questionnaire to assess the health-related QoL of cancer patients^11^. The questionnaire comprises five functional scales (physical, role, cognitive-emotional, and social), three symptom scales (fatigue, pain, and nausea and vomiting), and a global health and QoL scale. All items are scored on a 4-point Likert scale ranging from 1 “not at all” to 4 as “very much”, with the exception of two items in the global health/QoL scale, which uses modified 7-point linear analog scales [16]. The Korean EORTC-QLQ-C30 was developed by the EORTC group and was validated by Yun et al. in 2003 [17].

### Statistical analysis

Continuous data are indicated as median (interquartile range) or mean (standard deviation, SD) using Student’s t-test. Categorical data are presented as percentages and compared using a chi-squared test. The treatment effect or period effect was assessed by a paired t-test considering the cross-over design. A p-value less than 0.05 was accepted as the threshold to discriminate significant from non-significant. All statistical analyses were performed the SAS version 9.4 software (SAS Institute Inc., Cary, NC, USA).

## Results

### Clinical characteristics

**Fig. 1:**
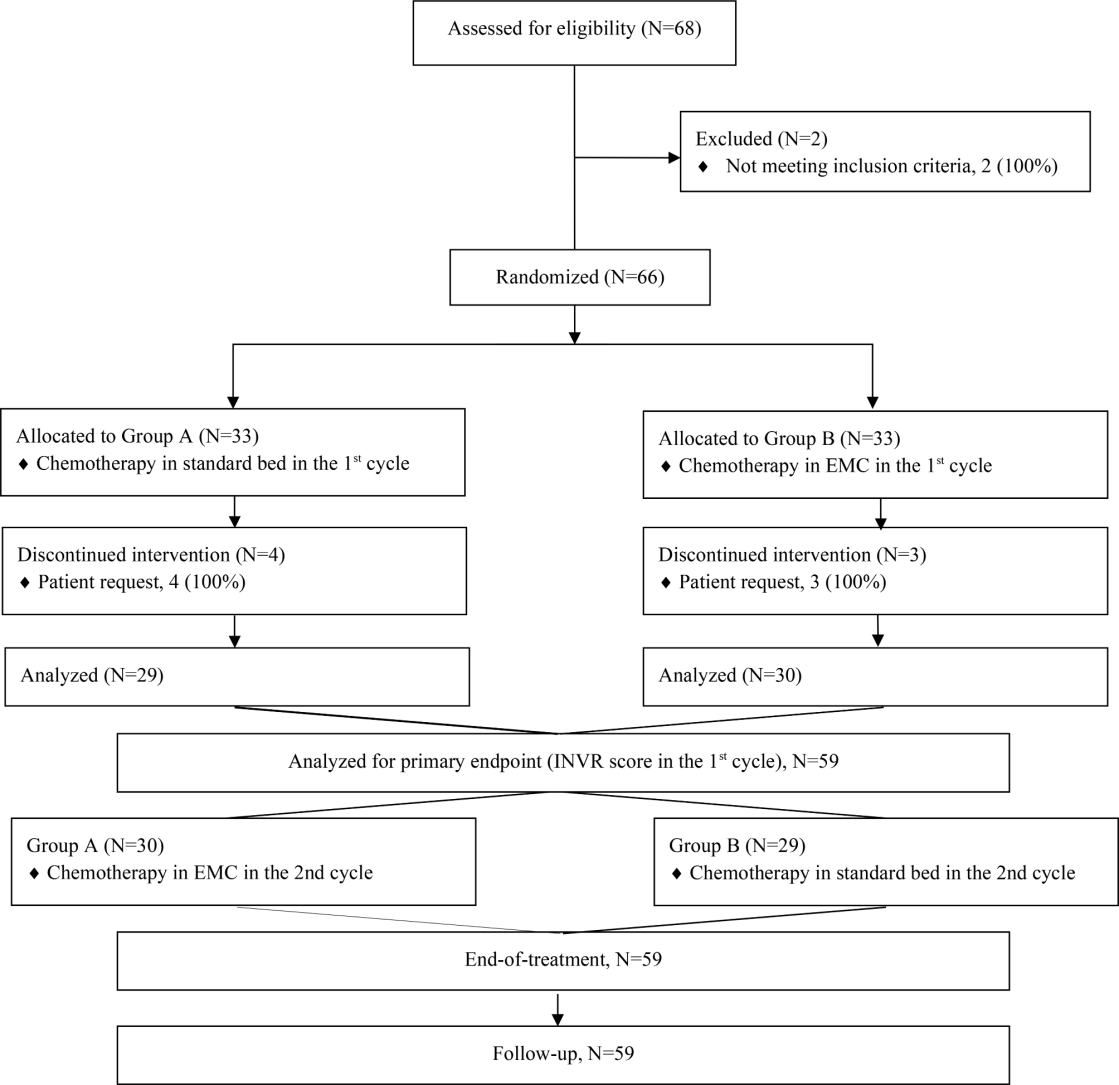
Study scheme. After screening 68 patients, a total of 66 of them were randomized. Participants who met inclusion criteria were randomized to Group A or Group B. Group A received their chemotherapy in general bed for the 1st cycle. Group B received chemotherapy in an EMC for the 1st cycle. Finally, 29 patients in Group A and 30 patients in Group B were analyzed for the results

The trial was conducted the initial registration on 01/07/2020 to the last patient’s final follow-up date on 08/09/2021. After screening 68 patients, 66 patients with cancer were enrolled in the study (Fig. 1). Among them, 59 patients completed the study protocol and were included in the analysis (per-protocol population, *N* = 59). Twenty-nine patients were assigned to Group A and 30 patients were assigned to Group B. The clinical characteristics of the patients are summarized in Table 1. The median age of the participants was 55 years (range: 52–68 years). The most common type of cancer was colorectal cancer (66.1%), followed by breast cancer (25.42%). Forty-three (72.88%) patients received MEC agents, mostly composed of FOLFIRI (irinotecan, 5-FU, and leucovorin) or FOLFOX (oxaliplatin, 5-FU, and leucovorin) for gastrointestinal cancer. Sixteen (27.12%) patients received HEC treatment including cisplatin or doxorubicin. There were no statistical differences between Groups A and B in terms of baseline clinical characteristics.

**Table 1:** Clinical characteristics

Clinical characteristics	Total (N = 59)	Group A (N = 29)	Group B (N = 30)	p-value
Age in years, median (IQR)	55 (52,68)	56 (52,62)	55 (53,68)	0.6496
Sex, n (%)				0.2184
Female	36 (61.02)	20 (68.97)	16 (53.33)	
Male	23 (38.98)	9 (31.03)	14 (46.67)	
Cancer types, n (%)				
Gastric cancer	3 (5.08)	2 (6.90)	1 (3.33)	0.5334
Colorectal cancer	39 (66.10)	19 (65.52)	20 (66.67)	0.9257
Breast cancer	15 (25.42)	6 (20.69)	9 (30.00)	0.4116
Others	2 (3.39)	2 (6.90)	0 (0.00)	0.1434
Chemotherapy, n (%)				0.9367
MEC	43 (72.88)	21 (72.41)	22 (73.33)	
HEC	16 (27.12)	8 (27.59)	8 (26.67)	
Baseline EORTC-QLQ-C30 score, mean (SD)	50.03 ± 14.01	47.00 ± 13.10	52.97 ± 14.44	0.1019
Baseline stress score, mean (SD)	13.90 ± 6.87	13.00 ± 7.22	14.77 ± 6.53	0.3288

IQR, interquartile range; MEC, moderate emetic risk chemotherapy; HEC, high emetic risk chemotherapy; SD, standard deviation

### CINV between the groups

The severity of acute CINV after the first treatment was the primary endpoint of this trial (Table 2). Patients in Group A received chemotherapy in the standard ward and served as the control group, whereas patients in Group B received treatment in an EMC and served as the intervention group. The mean INVR scores at cycle 1 day 2 (C1D2) were 2.76 ± 4.78 points in Group A and 3.63 ± 5.35 points in Group B (p-value = 0.5367). The CINV score of Group A was 1.48 ± 2.54 and Group B was 1.63 ± 2.59 (p-value = 0.0675). The differences were not statistically significant.

**Table 2:** Comparison of INVR (Index of Nausea, Vomiting, and Retching) score and CINV (Chemotherapy-induced nausea and vomiting) score between the groups in cycle 1 & 2

	Group A (N = 29)		Group B (N = 30)		p-value^(a)^	p-value^(b)^
	Mean ± SD	Median (IQR)	Mean ± SD	Median (IQR)		
**CINV**					0.0675	0.8148
Cycle 1	1.48 ± 2.54	0 [0, 2]	1.63 ± 2.59	0 [0, 3]		
Cycle 2	1.07 ± 1.71	0 [0, 2]	2.17 ± 2.68	1 [0, 5]		
**INVR**					0.5367	0.5367
Cycle 1	2.76 ± 4.78	0 [0, 5]	3.63 ± 5.35	0 [0, 7]		
Cycle 2	2.76 ± 4.27	0 [0, 4]	4.27 ± 5.23	2 [0, 9]		

(a) p-value by paired t-test

(b) p-value by period effect

In the 2nd chemotherapy cycle, when Group A received additive EMC therapy while Group B received standard care only, the median INVR score of Group A was 2.76 ± 4.27 and Group B was 4.27 ± 5.23 (p-value = 0.5367). The CINV score was 1.07 ± 1.71 in Group A and 2.17 ± 2.68 in Group B (p-value = 0.0675). The difference in INVR scores between the 1st and 2nd cycles (2nd cycle score – 1st cycle score) was 0 in Group A and + 0.64 in Group B. None of the differences were statistically significant.

### Complete response (CR) of CINV

Rescue medication and complete response (CR) rate to both acute and delayed CINV were assessed in each group. Rescue medications administered to patients during the trial are presented in Table 3. As per the protocol, 25 patients (86.2%) in Group A showed a CR in the acute phase of 1st cycle while 22 patients (73.3%) in Group B achieved a CR. Because of ethical issues, some patients with poor general condition were prescribed additive premedication and aprepitant for MEC, based on the investigators’ decision. When the analysis excluded patients who received additive premedication, the CR rate in the acute phase of 1st cycle in Group A was 85.0% and 72.0% in Group B (Table 3). Upgraded premedication, NEPA (netupitant/palonosetron), was also applied in the 2nd cycle when the patients presented with grade 3 CINV to MEC in the 1st cycle. Participants who received NEPA prescriptions, potentially impacting the calculation of the CR rate, numbered 9 in Group A and 5 in Group B. When excluding these populations, the CR in 2nd cycle was 80.0% in Group A and 76.0% in Group B (Table 3). There were no statistically significant differences between the groups at any time point.

**Table 3:** Comparison of complete response rate between groups in cycle 1 & 2

Total	Group A (N = 29)	Group B (N = 30)	p-value
Cycle 1 Day 2, n (%)	25 (86.2%)	22 (73.3%)	0.21974
Cycle 1 Day 6, n (%)	29 (100.0%)	29 (96.67%)	0.3214
Cycle 2 Day 2, n (%)	26 (89.7%)	23 (76.7%)	0.1837
Cycle 2 Day 6, n (%)	29 (100.0%)	30 (100.0%)	
**No other antiemetics**	**Group A** (*N* = 20)	**Group B** (*N* = 25)	**p-value**
Cycle 1 Day 2, n (%)	17 (85.0%)	18 (72.0%)	0.2973
Cycle 1 Day 6, n (%)	20 (100%)	24 (96.0%)	0.3657
Cycle 2 Day 2, n (%)	18 (80.0%)	19 (76.0%)	0.2222
Cycle 2 Day 6, n (%)	20 (100%)	25 (100.0%)	

### QoL assessment

**Fig. 2:**
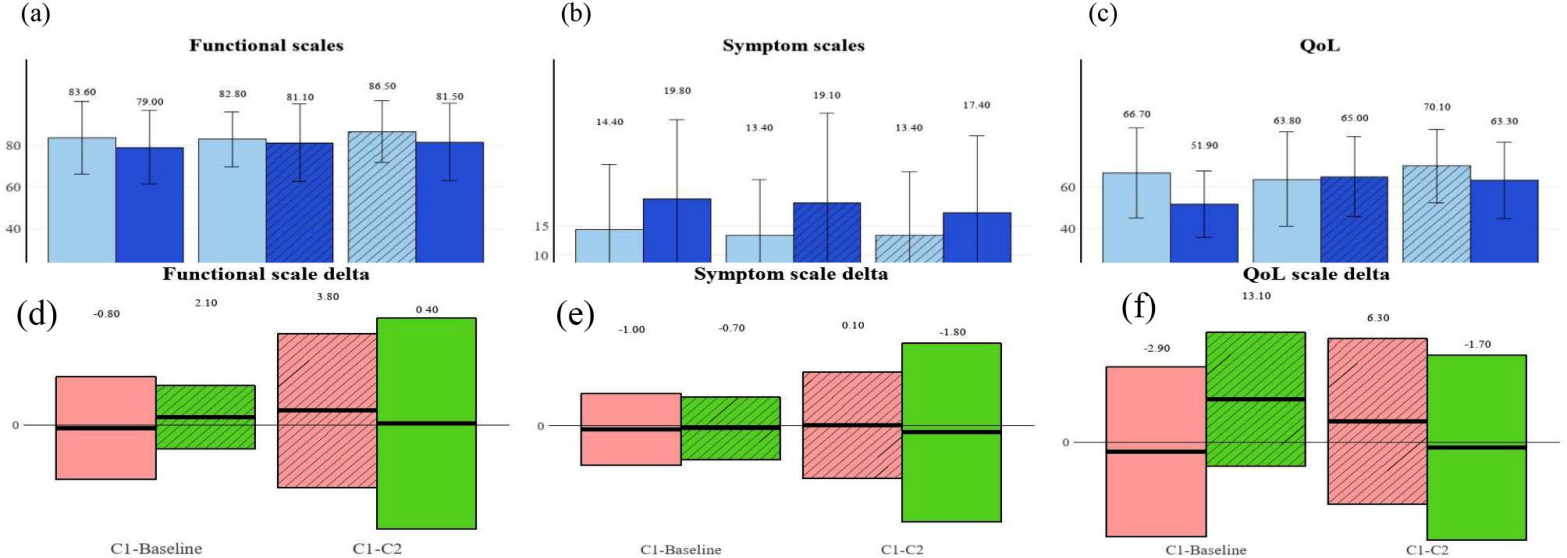
QoL assessment between groups. Tendency of EORTC-QLQ-C30 scales during the chemotherapy cycles. The hatch in the bar means receiving EMC. (**a**) Functional scales, (**b**) Symptom scales, and (**c**) QoL scale. Change of scales comparing with former score (**d**) delta of functional scale, (**e**) delta of symptom scales, and (**f**) delta of QoL scale

We assessed patient QoL using the EORTC-QLQ-C30 survey at three time points (before chemotherapy, after the 1st cycle, and after the 2nd cycle). A higher score indicates a better QoL and less distress. As visualized in Fig. 2 and summarized in Supplementary Table 1, there were differences in the baseline score between Groups A and B. Group A showed higher (better) functional scales and lower (worse) symptom scales than Group B (functional scales 83.6 vs. 78.96, respectively, p-value = 0.3181; symptom scales 14.41 vs. 19.83, respectively, p-value = 0.1163). QoL scores were significantly different between the two groups (66.67 vs. 51.94 for Group A vs. B, p-value = 0.0042). Functional and QoL scales of each group showed an increasing tendency, while symptom scales decreasing as treatment progressed through 2nd cycle (Fig. 2a-c). Considering the differences in baseline scale scores between groups, we analyzed changes in scales according to each cycle (Figure d-f). As a result, Group B, who received EMC in addition to the standard-of-care in the 1st cycle, showed significantly increase of QoL values compared with Group A (-2.87 vs. +13.06, respectively, p-value = 0.0108). Functional scales improved more and symptom scales decreased after the 1st cycle when the EMC intervention was applied (functional scales − 0.84 vs. +2.15 for Group A vs. B, p-value = 0.316; symptom scales − 0.97 vs. -0.68 for Group A vs. B, p-value = 0.9004). The scales improved more and worsened less in Group A than in Group B between the 2nd cycle and 1st cycle, but the differences were insignificant.

### Subgroup analysis; high-risk patients

**Fig. 3:**
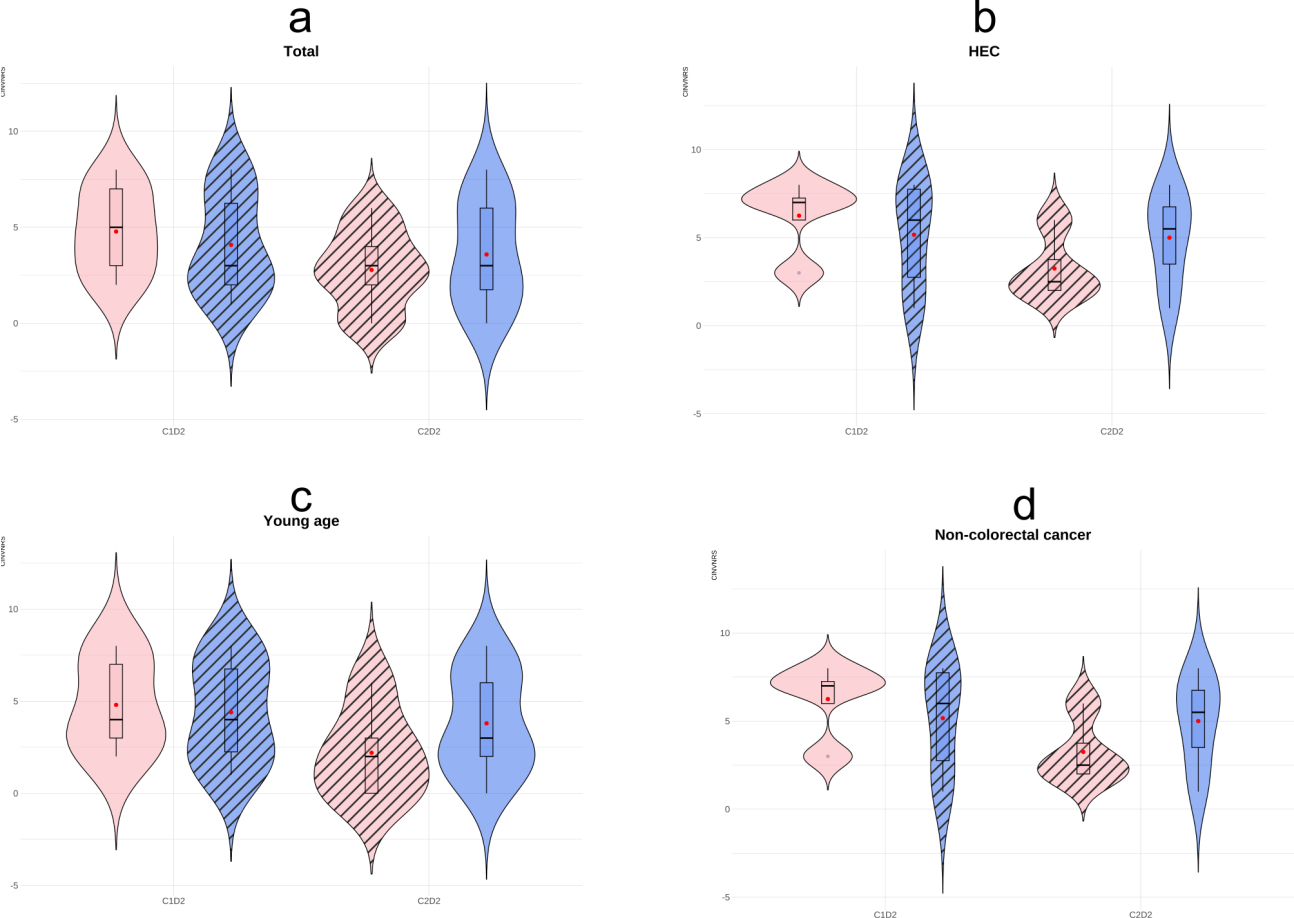
Subgroup analysis of CINV score in high-risk patients. Subgroup analysis conducted with patients who present INVR score of 3 or higher in the 1st cycle. The hatch in the bar means receiving EMC. (**a**) total high-risk subgroup, (**b**) patients who received HEC, (**c**) patients with younger age, and (**d**) patients with non-colorectal cancer

To explore whether EMCs would be effective in high-risk patients, we conducted a subgroup analysis. High-risk patients were defined as those who had an INVR score of ≥ 3 during the first cycle of chemotherapy. A total of 21 high-risk patients (9 in Group A and 12 in Group B) were analyzed, and the results are summarized in Fig. 3 and Supplementary Table 2. Although statistically insignificant, patients who received EMC therapy in addition to standard-of-care showed lower CINV scores than the control group in both cycles (1st cycle: 4.778 vs. 4.083; 2nd cycle: 2.778 vs. 3.583, p-value = 0.0902). Among the high-emetic risk subgroups, patients who received HEC (1st cycle 6.25 vs. 5.167 and 2nd cycle 3.25 vs. 5, p-value = 0.0495), younger age (1st cycle 4.8 vs. 4.4, 2nd cycle 2.2 vs. 3.8, p-value = 0.0108), and non-colorectal cancer patients (1st cycle 6.25 vs. 5.167 and 2nd cycle 3.28 vs. 5, p-value = 0.0495) presented statistically significant differences in CINV score according to additive EMC therapy.

### Safety analysis

All reported adverse events are summarized in Supplementary Table 3. Eighty adverse events were reported during this clinical trial. Among these, 15 (18.8%) were classified as grade 3 or 4. All adverse events were related to chemotherapy itself, and none of the study population was injured by the EMC or interrupted therapy due to adverse events. There were no instances of attrition due to toxicities during the study, as all participants completed the EMC protocol.

## Discussion

We conducted an open-label, randomized, cross-over phase II trial to assess the clinical efficacy of an EMC in preventing CINV. Fifty-nine patients receiving MEC or HEC for the first time were randomized and analyzed. Overall, no significant difference was observed in the prevention of CINV between patients treated with or without the EMC, in terms of INVR score (2.76 vs. 3.63, p-value = 0.5367) or CINV severity (1.48 vs. 1.63, p-value = 0.0675). However, QoL improvements were noted in patients receiving chemotherapy in the EMC, particularly in those undergoing HEC.

CINV, a major side effect of cancer treatment, can severely compromise patient QoL and compliance, potentially leading to treatment discontinuation. Both physiological and psychological factors influence CINV. A study in Europe found that patients with emotional distress and maladaptive coping experienced more severe CINV [18], highlighting the role of psychological vulnerability in CINV.

Despite numerous antiemetics, complete CINV remission remains elusive. NEPA, an oral drug combining an NK1 receptor antagonist and a 5HT3 receptor antagonist, has shown efficacy in HEC or MEC patients [19]. In a recent phase 3 clinical trial, a combination of oral dexamethasone and NEPA, which is considered the most efficacious regimen for preventing CINV, showed a CR of 73.8% during the overall phase in patients received HEC [20]. The most commonly reported adverse events were constipation (8.0%) and hiccups (2.7%) [20]. However, even the most effective antiemetic combinations cannot perfectly control CINV.

To address this unmet clinical need, many integrative therapies have been investigated and tested. Among the complementary measures, acupuncture and massage therapy are key alternative methods that have been shown to reduce symptoms [21]. Although the precise mechanism of action has not been fully elucidated, massage therapy is preferred due to its non-invasiveness, and it has been shown to relieve multiple distressing symptoms in cancer patients, including pain, anxiety, fatigue, and nausea [22]. Music therapy, encompassing interactive (e.g., improvisation, singing) and receptive (e.g., listening to music) techniques, significantly benefits cancer patients by boosting mood, alleviating stress, pain, and anxiety, and fostering relaxation [23]. As a crucial component of supportive cancer care, it not only assists during treatment but also sets the stage for successful rehabilitation, ultimately improving wellness, physical and emotional health, and quality of life. Considering the use of New Age style music in our study and the variety of music types in previous research [24], it’s necessary to explore which type of music is the most effective.

In our study, we combined mechanical massage with relaxing music using EMCs. This approach was as effective as physiotherapy in controlling pain and improving QoL and satisfaction, and more cost-effective than manual massage [25]. EMCs have also been reported to manage chronic stress effectively [13] and reduce cortisol levels more than mental training [26], suggesting their potential as a paramedical option in modern cancer care.

However, our study didn’t meet the primary endpoint of a statistically lower INVR score for C1D2 with EMC therapy. This may be due to the subjective nature of CINV reporting and the novel experience of chemotherapy for participants, complicating symptom description. Ethical considerations required the early prescription of antiemetics, affecting the calculation of CR rates. Our phase II trial’s small size and lack of patient stratification by clinical characteristics also limited our findings.

Subgroup analysis, however, indicated EMC’s potential efficacy in high-risk patients. Along with the chemotherapeutic agent itself, several patient-related factors determine the degree of CINV. A history of CINV, expectancy of CINV, female sex, younger age, history of morning sickness, and low alcohol consumption are important risk factors for CINV [27]. Prior research indicated that for every year younger in age, the likelihood of experiencing CINV rises by 4%. Female patients face a substantially greater risk compared to male patients, marked by an odds ratio of 2.79. Additionally, having a lower risk associated with alcohol consumption corresponds to an odds ratio of 1.94, while a history of morning sickness correlates with an elevated odds ratio of 1.97. Patients with a history of CINV at earlier treatment were at a significantly higher risk of CINV (summary odds ratio = 1.67, 95% CI 1.41–1.99) [27]. Having had nausea in the previous cycle could be a risk factor not only for anticipatory nausea but also for acute and delayed nausea [28]. With this background, we defined high-risk patients as those who presented with severe nausea (INVR score ≥ 3) in the first cycle. Patients who received HEC, younger patients, and non-colorectal cancer patients presented with less severe CINV when an EMC was used. Appropriate integrated medicine, in addition to standard treatment, could be an effective and safe option for high-risk CINV patients.

Our study had several limitations. We enrolled patients who received HEC or MEC, regardless of the tumor type or other clinical factors, including stage. Therefore, participants’ characteristics were heterogeneous. Additive antiemetic agents were administered based on patients’ complaints of symptoms and investigators’ decisions, resulting in a bias in the CR rate. We adopted the standard massage mode inherent in the commercial EMC; whether this is optimal is unknown due to the lack of previous research. Due to the varying body types of participants, it was challenging to pinpoint the exact massage targets of the EMC. Further research is needed to determine the optimal mode for cancer patients. In addition, as a phase II clinical trial with a sample size of less than 70 participants, the statistical meaning should be interpreted with caution.

To our knowledge, this is the first randomized trial assessing EMC’s effect on CINV. While it didn’t conclusively prove EMC’s CINV-reducing effect, high-risk patients tended to experience lower symptom severity and improved QoL with EMC treatment. The rising demand for caregiving and healthcare costs suggests that massage chairs could offer a viable alternative. Collecting more real-world data and further studies are necessary to minimize chemotherapy’s side effects using both medication and medical devices.

### Electronic supplementary material

Below is the link to the electronic supplementary material.

Supplementary Material 1

## Data Availability

The datasets used and/or analysed during the current study available from the corresponding author on reasonable request.
