# Supplementary material for: The clinical effect of an electric massage chair on chemotherapy-induced nausea and vomiting in cancer patients: randomized phase II cross-over trial
Source: BMC Complement Med Ther. 2024 Apr 19;24:163. doi: 10.1186/s12906-024-04464-8 (PMC11027524; doi:10.1186/s12906-024-04464-8)
Supplement: Supplementary file 1 — Supplementary Material 1 [file 12906_2024_4464_MOESM1_ESM.docx]

Supplementary table 1. Quality of life assessment between groups

| **EORTC-QLQ-C30 score** | | | | | |
| --- | --- | --- | --- | --- | --- |
|  | **Group A (N=29)** | | **Group B (N=30)** | | **p-value** |
| **Baseline** | Mean (SD) | Median [IQR] | Mean (SD) | Median [IQR] | 0.1019 |
| Functional scales | 83.6 (17.26) | 88.89  [77.78, 95.56] | 78.96 (18.09) | 82.22  [73.33, 93.33] | 0.3181 |
| Symptom scales | 14.41 (11.41) | 12.82  [5.13, 23.08] | 19.83 (14.44) | 16.67  [10.26, 28.21] | 0.1163 |
| QoL | 66.67 (21.48) | 66.67  [50, 83.33] | 51.94 (16.18) | 50  [41.67, 66.67] | 0.0042 |
| **After 1^st^ cycle** | Mean (SD) | Median [IQR] | Mean (SD) | Median [IQR] | 0.3971 |
| Functional scales | 82.76 (13.3) | 84.44  [75.56, 93.33] | 81.11 (18.5) | 85.56  [75.56, 93.33] | 0.6968 |
| Symptom scales | 13.44 (9.8) | 10.26  [5.13, 17.95] | 19.15 (15.69) | 15.38  [10.26, 25.64] | 0.0991 |
| QoL | 63.79 (22.52) | 66.67  [50, 83.33] | 65 (19.38) | 66.67  [50, 75] | 0.826 |
| **After 2^nd^ cycle** | Mean (SD) | Median [IQR] | Mean (SD) | Median [IQR] | 0.1783 |
| Functional scales | 86.51 (14.76) | 91.11  [80, 97.78] | 81.48 (18.16) | 84.44  [73.33, 91.11] | 0.2487 |
| Symptom scales | 13.35 (11.12) | 10.26  [5.13, 17.95] | 17.35 (13.14) | 12.82  [10.26, 23.08] | 0.2128 |
| QoL | 70.11 (17.61) | 66.67  [58.33, 83.33] | 63.33 (18.52) | 54.17  [50, 83.33] | 0.1552 |
| **Change of EORTC-QLQ-C30 score** | | | | | |
|  | **Group A (N=29)** | | **Group B (N=30)** | | **p-value** |
| **After 1^st^ cycle - Baseline** | Mean (SD) | Median [IQR] | Mean (SD) | Median [IQR] |  |
| Functional scales | -0.84 (13.64) | -2 [-7, 4] | 2.15 (8.53) | 2 [-2, 7] | 0.3196 |
| Symptom scales | -0.97 (9.38) | -3 [-5, 5] | -0.68 (8.25) | 0 [-5, 3] | 0.9004 |
| QoL | -2.87 (26.00) | 0 [-17, 8] | 13.06 (20.14) | 8 [0, 17] | 0.0108 |
| **After 2nd cycle – After 1st cycle** | Mean (SD) | Median [IQR] | Mean (SD) | Median [IQR] |  |
| Functional scales | 3.75 (20.27) | 0 [-7, 16] | 0.37 (27.53) | -2 [-9, 11] | 0.5939 |
| Symptom scales | -0.09 (13.92) | 0 [-8, 5] | -1.79 (23.07) | 0 [-8, 13] | 0.7314 |
| QoL | 6.32 (25.26) | 0 [-17, 25] | -1.67 (27.80) | -8 [-17, 17] | 0.2534 |

Supplementary table 2. Subgroup analysis of CINV scores in high-risk patients

|  | **Group A (N=9)** | | **Group B (N=12)** | | **p-value^(a)^** | **p-value^(b)^** |
| --- | --- | --- | --- | --- | --- | --- |
|  | Mean (SD) | Median [IQR] | Mean (SD) | Median [IQR] |  |  |
| **Total** | N=9 | | N=12 | | 0.0902 | 0.0078 |
| Cycle 1 | 4.778 (2.224) | 5 [3, 7] | 4.083 (2.610) | 3 [2, 6.5] |  |  |
| Cycle 2 | 2.778 (2.048) | 3 [2, 4] | 3.583 (2.746) | 3 [1.5, 6] |  |  |
| **Age < 57.7** | N=5 | | N=10 | | 0.0108 | 0.0004 |
| Cycle 1 | 4.8 (2.588) | 4 [3, 7] | 4.4 (2.757) | 4 [2, 7] |  |  |
| Cycle 2 | 2.2 (2.490) | 2 [0, 3] | 3.8 (2.741) | 3 [2, 6] |  |  |
| **Age > 57.7** | N=4 | | N=2 | | 0.6883 | 0.6883 |
| Cycle 1 | 4.75 (2.062) | 5 [3.5, 6] | 2.5 (0.707) | 2.5 [2, 3] |  |  |
| Cycle 2 | 3.5 (1.291) | 3.5 [2.5, 4.5] | 2.5 (3.536) | 2.5 [0, 5] |  |  |
| **MEC** | N=5 | | N=6 | | 0.7541 | 0.1069 |
| Cycle 1 | 3.6 (1.517) | 4 [2, 5] | 3 (1.673) | 3 [2, 3] |  |  |
| Cycle 2 | 2.4 (2.302) | 3 [0, 4] | 2.167 (2.229) | 2 [0, 3] |  |  |
| **HEC** | N=4 | | N=6 | | 0.0495 | 0.0324 |
| Cycle 1 | 6.25 (2.217) | 7 [5, 7.5] | 5.167 (3.061) | 6 [2, 8] |  |  |
| Cycle 2 | 3.25 (1.893) | 2.5 [2, 4.5] | 5 (2.608) | 3.25 (1.893) |  |  |
| **Female** | N=7 | | N=9 | | 0.0695 | 0.0453 |
| Cycle 1 | 4.857 (2.545) | 5 [2, 7] | 4.556 (0.707) | 5 [2, 7] |  |  |
| Cycle 2 | 2.857 (1.864) | 3 [2, 4] | 4.444 (2.506) | 5 [2, 6] |  |  |
| **Male** | N=2 | | N=3 | | 0.8703 | 0.1456 |
| Cycle 1 | 4.5 (0.707) | 4.5 [4, 5] | 2.667 (0.577) | 3 [2, 3] |  |  |
| Cycle 2 | 2.5 (3.536) | 2.5 [0, 5] | 1 (1.732) | 0 [0, 3] |  |  |
| **Colorectal cancer** | N=5 | | N=6 | | 0.7541 | 0.0324 |
| Cycle 1 | 3.6 (1.517) | 4 [2, 5] | 3 (1.673) | 3 [2, 3] |  |  |
| Cycle 2 | 2.4 (2.302) | 3 [0, 4] | 2.167 (2.229) | 2 [0, 3] |  |  |
| **Non-colorectal cancer** | N=4 | | N=6 | | 0.0495 | 0.0324 |
| Cycle 1 | 6.25 (2.217) | 7 [5, 7.5] | 5.167 (3.061) | 6 [2, 8] |  |  |
| Cycle 2 | 3.25 (1.893) | 2.5 [2, 4.5] | 5 (2.608) | 5.5 [3, 7] |  |  |

Supplementary table 3. Adverse events collected during the trial

| Adverse events | Total (N=59) | |
| --- | --- | --- |
|  | Any grade | Grade 3 or 4 |
| Total, n (%) | 80 (100.0%) | 15 (100.0%) |
| Abdominal pain | 1 (1.3%) | 0 (0%) |
| Alopecia | 1 (1.3%) | 0 (0%) |
| Anorexia, n (%) | 7 (8.8%) | 0 (0%) |
| Constipation | 14 (17.5%) | 1 (6.7%) |
| Diarrhea | 22 (27.5%) | 5 (33.3%) |
| Dyspepsia | 1 (1.3%) | 0 (0%) |
| Fever | 2 (2.5%) | 0 (0%) |
| General weakness | 3 (3.8%) | 0 (0%) |
| Headache | 8 (10.0%) | 0 (0%) |
| Heartburn | 2 (1.3%) | 0 (0%) |
| Hepatitis | 1 (1.3%) | 0 (0%) |
| Mucositis | 3 (3.8%) | 0 (0%) |
| Myalgia | 2 (1.3%) | 0 (0%) |
| Decreased neutrophil count | 9 (11.3%) | 9 (60.0%) |
| Paresthesia | 2 (1.3%) | 0 (0%) |
| Skin rash | 2 (1.3%) | 0 (0%) |
